# 5-Cyclo­hexyl-2-(4-fluoro­phen­yl)-3-phenyl­sulfinyl-1-benzo­furan

**DOI:** 10.1107/S1600536813022678

**Published:** 2013-08-21

**Authors:** Hong Dae Choi, Pil Ja Seo, Uk Lee

**Affiliations:** a Department of Chemistry, Dongeui University, San 24 Kaya-dong, Busanjin-gu, Busan 614-714, Republic of Korea; b Department of Chemistry, Pukyong National University, 599-1 Daeyeon 3-dong, Nam-gu, Busan 608-737, Republic of Korea

## Abstract

The asymmetric unit of the title compound, C_26_H_23_FO_2_S, contains two independent mol­ecules (*A* and *B*), in both of which the cyclo­hexyl ring adopts a chair conformation. The benzo­furan ring systems, the 4-fluoro­phenyl and phenyl rings are essentially planar, with r.m.s. deviations of 0.008 (1), 0.002 (1) and 0.003 (1) Å, respectively, for mol­ecule *A*, and 0.016 (1), 0.004 (1) and 0.002 (1) Å, respectively, for mol­ecule *B*. The dihedral angles between the benzo­furan ring system and the pendant 4-fluoro­phenyl and phenyl rings are 12.3 (7) and 85.42 (4)°, respectively, for mol­ecule *A*, and 39.67 (6) and 72.17 (4)°, respectively, for mol­ecule *B*. In the crystal, mol­ecules are linked by weak C—H⋯O and C—H⋯π inter­actions, resulting in a three-dimensional network.

## Related literature

For background information and the crystal structures of related compounds, see: Choi *et al.* (2011 ▶, 2012 ▶); Seo *et al.* (2011 ▶).
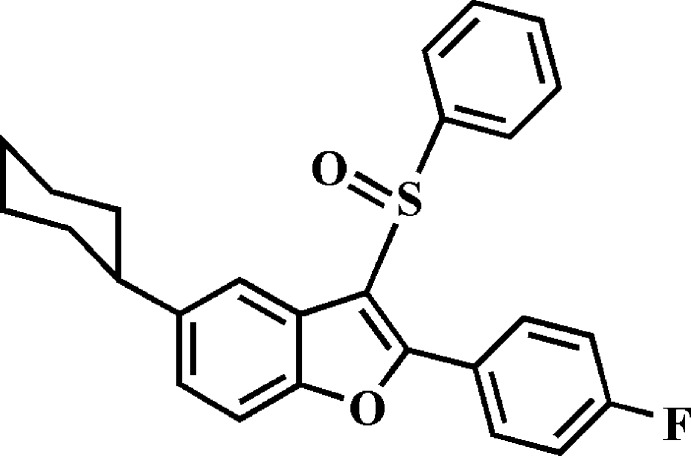


## Experimental

### 

#### Crystal data

C_26_H_23_FO_2_S
*M* *_r_* = 418.50
Triclinic, 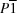

*a* = 9.1536 (2) Å
*b* = 12.6562 (2) Å
*c* = 19.3939 (4) Å
α = 84.674 (1)°
β = 79.667 (1)°
γ = 72.405 (1)°
*V* = 2105.15 (7) Å^3^
*Z* = 4
Mo *K*α radiation
μ = 0.18 mm^−1^
*T* = 173 K
0.38 × 0.27 × 0.25 mm

#### Data collection

Bruker SMART APEXII CCD diffractometer
Absorption correction: multi-scan (*SADABS*; Bruker, 2009 ▶) *T*_min_ = 0.702, *T*_max_ = 0.746
36372 measured reflections
9183 independent reflections
7799 reflections with *I* > 2σ(*I*)
*R*_int_ = 0.028

#### Refinement

*R*[*F* ^2^ > 2σ(*F* ^2^)] = 0.037
*wR*(*F* ^2^) = 0.100
*S* = 1.03
9183 reflections
541 parameters
H-atom parameters constrained
Δρ_max_ = 0.34 e Å^−3^
Δρ_min_ = −0.35 e Å^−3^

### 

Data collection: *APEX2* (Bruker, 2009 ▶); cell refinement: *SAINT* (Bruker, 2009 ▶); data reduction: *SAINT*; program(s) used to solve structure: *SHELXS97* (Sheldrick, 2008 ▶); program(s) used to refine structure: *SHELXL97* (Sheldrick, 2008 ▶); molecular graphics: *ORTEP-3 for Windows* (Farrugia, 2012 ▶) and *DIAMOND* (Brandenburg 1998 ▶); software used to prepare material for publication: *SHELXL97*.

## Supplementary Material

Crystal structure: contains datablock(s) I. DOI: 10.1107/S1600536813022678/mw2115sup1.cif

Structure factors: contains datablock(s) I. DOI: 10.1107/S1600536813022678/mw2115Isup2.hkl

Supplementary material file. DOI: 10.1107/S1600536813022678/mw2115Isup3.cml

Additional supplementary materials:  crystallographic information; 3D view; checkCIF report

## Figures and Tables

**Table 1:** Hydrogen-bond geometry (Å, °) *Cg*1 and *Cg*2 are the centroids of the C41–C46 4-fluoro­phenyl ring and the C2–C7 benzene ring, respectively.

*D*—H⋯*A*	*D*—H	H⋯*A*	*D*⋯*A*	*D*—H⋯*A*
C10—H10*B*⋯O2^i^	0.99	2.47	3.384 (2)	153
C22—H22⋯O2^i^	0.95	2.42	3.2365 (19)	144
C19—H19⋯O4^ii^	0.95	2.44	3.3442 (19)	159
C40—H40*B*⋯*Cg*1^iii^	0.99	2.84	3.763 (2)	155
C45—H45⋯*Cg*2	0.95	2.82	3.602 (2)	140
C50—H50⋯*Cg*1^iv^	0.95	2.80	3.619 (2)	144

Symmetry codes: (i) 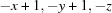; (ii) 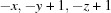; (iii) 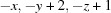; (iv) 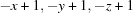.

**Fig. 1.**
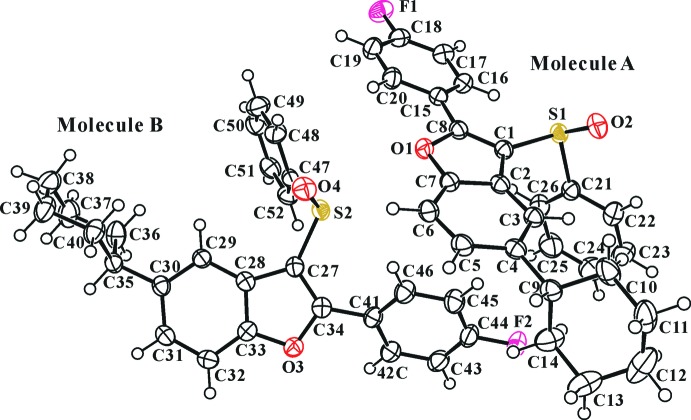
The molecular structure of the title compound with the atom numbering scheme. Displacement ellipsoids are drawn at the 50% probability level. H atoms are presented as small spheres of arbitrary radius.

**Fig. 2.**
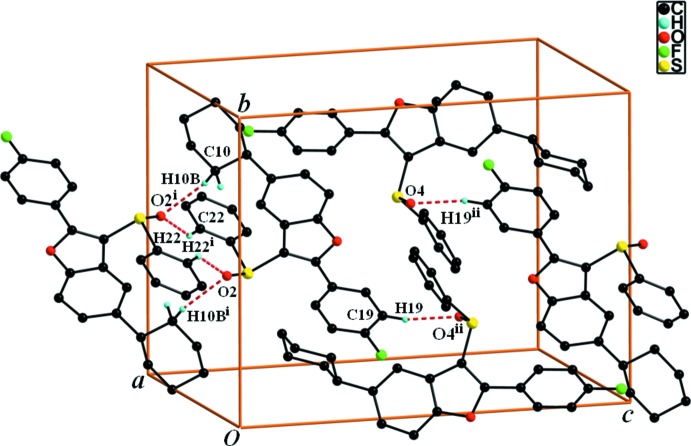
A view of the C—H···O interactions (dotted lines) in the crystal structure of the title compound. H atoms non-participating in hydrogen-bonding were omitted for clarity. [Symmetry codes: (i) - x + 1, - y + 1, - z; (ii) - x, - y + 1, - z + 1.]

**Fig. 3.**
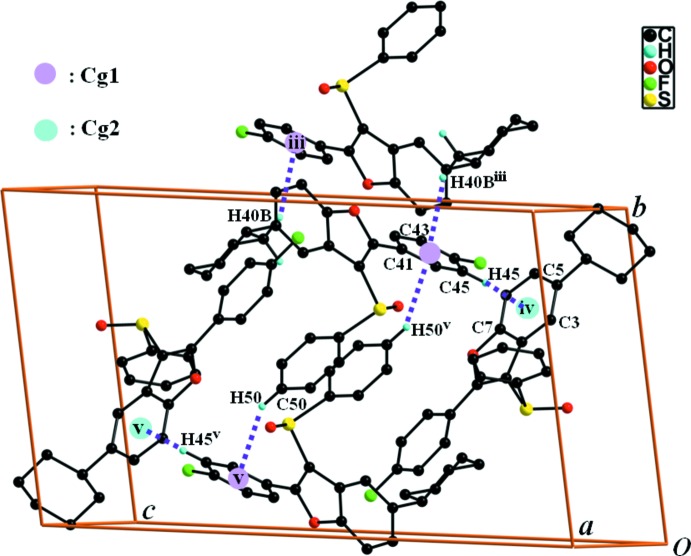
A view of the C—H···π interactions (dotted lines) in the crystal structure of the title compound. H atoms non-participating in hydrogen-bonding were omitted for clarity. [Symmetry codes: (iii) - x, 2- y + 2, - z + 1; (iv) x, y, z; (v) - x + 1, - y + 1, - z + 1.]

## supplementary crystallographic information

### 1. Comment

As a part of our continuing study of 2-(4-fluorophenyl)-3-phenylsulfinyl-1-benzofuran derivatives containing chloro (Choi *et al.*, 2011), bromo (Seo *et al.*, 2011), and iodo (Choi *et al.*, 2012) groups in 5-position, we report here the crystal structure of the title compound which crystallizes with two independent molecules, A & B, in the asymmetric unit.

In the title compound (Fig. 1), the cyclohexyl ring of both molecules adopts a chair conformation. The benzofuran ring system is essentially planar, with a mean deviation of 0.008 (1) and 0.016 (1) Å, for A and B, respectively, from the least-squares plane defined by the nine constituent atoms. The 4-fluorophenyl and phenyl rings are essentially planar, with mean deviations of 0.002 (1) and 0.003 (1) Å for molecule A, and 0.004 (1) and 0.002 (1) Å for molecule B, respectively, from the least-squares plane defined by the six constituent atoms. The dihedral angles formed by the benzofuran ring system and the pendant 4-fluorophenyl and phenyl rings are 12.31 (7) and 85.42 (4)° in molecule A, and 39.67 (6) and 72.17 (4)° in molecule B, respectively. In the crystal packing, molecules are connected by weak C—H···O hydrogen bonds (Fig. 2 and Table 2) and C—H···π interactions (Fig. 3 and Table 2, Cg1 and Cg2 are the centroids of the C41–C46 4-fluorophenyl ring and the C2–C7 benzene ring, respectively), resulting in a three-dimensional network.

### 2. Experimental

3-Chloroperoxybenzoic acid (77%, 202 mg, 0.9 mmol) was added in small portions to a stirred solution of 5-cyclohexyl-2-(4-fluorophenyl)-3-phenylsulfanyl-1-benzofuran (322 mg, 0.8 mmol) in dichloromethane (40 mL) at 273 K. After being stirred at room temperature for 4h, the mixture was washed with saturated sodium bicarbonate solution and the organic layer was separated, dried over magnesium sulfate, filtered and concentrated at reduced pressure. The residue was purified by column chromatography (hexane–ethyl acetate, 2:1 v/v) to afford the title compound as a colorless solid [yield 63%, m.p. 403–404 K; *R*_f_ = 0.78 (hexane–ethyl acetate, 2:1 v/v)]. Single crystals suitable for X-ray diffraction were prepared by slow evaporation of a solution of the title compound in acetone at room temperature.

### 3. Refinement

All H atoms were positioned geometrically and refined using a riding model with C—H = 0.95 Å for aryl, 1.00 Å for methine, 0.99 Å for methylene and 0.98 Å for methyl H atoms, respectively. Uiso(H) = 1.2Ueq(C) for aryl, methine and methylene, and 1.5Ueq(C) for methyl H atoms The positions of methyl hydrogens were optimized rotationally.

### Crystal data

**Table:**

C_26_H_23_FO_2_S	*Z* = 4
*M**_r_* = 418.50	*F*(000) = 880
Triclinic, *P*1	*D*_x_ = 1.320 Mg m^−^^3^
Hall symbol: -P 1	Melting point: 403 K
*a* = 9.1536 (2) Å	Mo *K*α radiation, λ = 0.71073 Å
*b* = 12.6562 (2) Å	Cell parameters from 9956 reflections
*c* = 19.3939 (4) Å	θ = 2.5–28.3°
α = 84.674 (1)°	µ = 0.18 mm^−^^1^
β = 79.667 (1)°	*T* = 173 K
γ = 72.405 (1)°	Block, colourless
*V* = 2105.15 (7) Å^3^	0.38 × 0.27 × 0.25 mm

### Data collection

**Table:**

Bruker SMART APEXII CCD diffractometer	9183 independent reflections
Radiation source: rotating anode	7799 reflections with *I* > 2σ(*I*)
Graphite multilayer monochromator	*R*_int_ = 0.028
Detector resolution: 10.0 pixels mm^-1^	θ_max_ = 27.0°, θ_min_ = 2.1°
φ and ω scans	*h* = −11→11
Absorption correction: multi-scan (*SADABS*; Bruker, 2009)	*k* = −16→16
*T*_min_ = 0.702, *T*_max_ = 0.746	*l* = −24→24
36372 measured reflections	

### Refinement

**Table:**

Refinement on *F*^2^	Primary atom site location: structure-invariant direct methods
Least-squares matrix: full	Secondary atom site location: difference Fourier map
*R*[*F*^2^ > 2σ(*F*^2^)] = 0.037	Hydrogen site location: difference Fourier map
*wR*(*F*^2^) = 0.100	H-atom parameters constrained
*S* = 1.03	*w* = 1/[σ^2^(*F*_o_^2^) + (0.0483*P*)^2^ + 0.7052*P*] where *P* = (*F*_o_^2^ + 2*F*_c_^2^)/3
9183 reflections	(Δ/σ)_max_ = 0.001
541 parameters	Δρ_max_ = 0.34 e Å^−^^3^
0 restraints	Δρ_min_ = −0.35 e Å^−^^3^

### Special details

**Table:**

Geometry. All esds (except the esd in the dihedral angle between two l.s. planes) are estimated using the full covariance matrix. The cell esds are taken into account individually in the estimation of esds in distances, angles and torsion angles; correlations between esds in cell parameters are only used when they are defined by crystal symmetry. An approximate (isotropic) treatment of cell esds is used for estimating esds involving l.s. planes.
Refinement. Refinement of F^2^ against ALL reflections. The weighted R-factor wR and goodness of fit S are based on F^2^, conventional R-factors R are based on F, with F set to zero for negative F^2^. The threshold expression of F^2^ > 2sigma(F^2^) is used only for calculating R-factors(gt) etc. and is not relevant to the choice of reflections for refinement. R-factors based on F^2^ are statistically about twice as large as those based on F, and R- factors based on ALL data will be even larger.

### Fractional atomic coordinates and isotropic or equivalent isotropic displacement parameters (Å^2^)

**Table:**

	*x*	*y*	*z*	*U*_iso_*/*U*_eq_	
S1	0.51941 (4)	0.39671 (3)	0.142600 (18)	0.02863 (9)	
F1	0.45285 (11)	0.11763 (8)	0.46866 (5)	0.0447 (2)	
O1	0.18092 (11)	0.54650 (8)	0.29154 (5)	0.0306 (2)	
O2	0.46030 (13)	0.40426 (9)	0.07538 (6)	0.0395 (3)	
C1	0.36986 (15)	0.48459 (11)	0.20035 (7)	0.0267 (3)	
C2	0.27527 (15)	0.59273 (11)	0.17968 (7)	0.0268 (3)	
C3	0.27759 (16)	0.66238 (11)	0.11966 (7)	0.0282 (3)	
H3	0.3544	0.6402	0.0796	0.034*	
C4	0.16491 (16)	0.76520 (11)	0.11967 (8)	0.0291 (3)	
C5	0.05089 (17)	0.79447 (12)	0.17950 (8)	0.0335 (3)	
H5	−0.0262	0.8642	0.1787	0.040*	
C6	0.04609 (17)	0.72625 (12)	0.23941 (8)	0.0336 (3)	
H6	−0.0318	0.7471	0.2793	0.040*	
C7	0.16126 (16)	0.62613 (11)	0.23777 (7)	0.0287 (3)	
C8	0.30912 (15)	0.46032 (11)	0.26738 (7)	0.0277 (3)	
C9	0.16859 (16)	0.84687 (11)	0.05791 (8)	0.0305 (3)	
H9	0.0613	0.8989	0.0598	0.037*	
C10	0.2120 (2)	0.79323 (14)	−0.01261 (9)	0.0507 (5)	
H10A	0.1345	0.7553	−0.0175	0.061*	
H10B	0.3143	0.7365	−0.0146	0.061*	
C11	0.2190 (3)	0.87806 (15)	−0.07329 (9)	0.0570 (5)	
H11A	0.2560	0.8392	−0.1180	0.068*	
H11B	0.1134	0.9287	−0.0752	0.068*	
C12	0.3265 (3)	0.9450 (2)	−0.06525 (12)	0.0708 (7)	
H12A	0.4341	0.8955	−0.0683	0.085*	
H12B	0.3240	1.0020	−0.1040	0.085*	
C13	0.2791 (3)	1.00101 (18)	0.00423 (11)	0.0609 (5)	
H13A	0.1751	1.0555	0.0056	0.073*	
H13B	0.3536	1.0416	0.0090	0.073*	
C14	0.2753 (2)	0.91600 (15)	0.06525 (10)	0.0477 (4)	
H14A	0.3816	0.8664	0.0668	0.057*	
H14B	0.2384	0.9549	0.1099	0.057*	
C15	0.34829 (16)	0.36826 (11)	0.31857 (7)	0.0283 (3)	
C16	0.49049 (16)	0.28528 (12)	0.30941 (8)	0.0313 (3)	
H16	0.5640	0.2873	0.2684	0.038*	
C17	0.52526 (17)	0.20011 (12)	0.35957 (8)	0.0337 (3)	
H17	0.6216	0.1434	0.3532	0.040*	
C18	0.41714 (18)	0.19941 (12)	0.41886 (8)	0.0335 (3)	
C19	0.27566 (19)	0.27905 (13)	0.43010 (8)	0.0377 (3)	
H19	0.2029	0.2760	0.4713	0.045*	
C20	0.24248 (18)	0.36376 (13)	0.37965 (8)	0.0347 (3)	
H20	0.1458	0.4200	0.3867	0.042*	
C21	0.65459 (16)	0.47748 (11)	0.13046 (7)	0.0298 (3)	
C22	0.70074 (19)	0.51549 (14)	0.06361 (8)	0.0397 (3)	
H22	0.6564	0.5044	0.0252	0.048*	
C23	0.8142 (2)	0.57062 (16)	0.05396 (11)	0.0545 (5)	
H23	0.8468	0.5985	0.0085	0.065*	
C24	0.8794 (2)	0.58498 (17)	0.10984 (12)	0.0577 (5)	
H24	0.9577	0.6217	0.1025	0.069*	
C25	0.8316 (2)	0.54631 (17)	0.17639 (11)	0.0526 (5)	
H25	0.8764	0.5571	0.2148	0.063*	
C26	0.71881 (18)	0.49197 (14)	0.18720 (8)	0.0391 (3)	
H26	0.6857	0.4649	0.2328	0.047*	
S2	0.20524 (4)	0.67170 (3)	0.453110 (18)	0.03051 (9)	
F2	0.64505 (14)	0.83089 (9)	0.17479 (5)	0.0610 (3)	
O3	0.25741 (11)	0.96430 (8)	0.47026 (5)	0.0311 (2)	
O4	0.04149 (12)	0.67966 (9)	0.44733 (6)	0.0395 (3)	
C27	0.20969 (16)	0.79849 (12)	0.48159 (7)	0.0289 (3)	
C28	0.14132 (15)	0.85432 (11)	0.54677 (7)	0.0275 (3)	
C29	0.06147 (16)	0.82926 (12)	0.61151 (7)	0.0289 (3)	
H29	0.0364	0.7611	0.6196	0.035*	
C30	0.01899 (16)	0.90582 (12)	0.66422 (8)	0.0296 (3)	
C31	0.05450 (17)	1.00664 (12)	0.65041 (8)	0.0325 (3)	
H31	0.0240	1.0584	0.6866	0.039*	
C32	0.13222 (17)	1.03442 (12)	0.58634 (8)	0.0333 (3)	
H32	0.1544	1.1035	0.5775	0.040*	
C33	0.17505 (16)	0.95539 (11)	0.53641 (7)	0.0287 (3)	
C34	0.27741 (16)	0.86767 (12)	0.43847 (7)	0.0296 (3)	
C35	−0.06404 (16)	0.88143 (12)	0.73604 (8)	0.0315 (3)	
H35	−0.0885	0.9490	0.7639	0.038*	
C36	0.0381 (2)	0.78558 (16)	0.77551 (8)	0.0457 (4)	
H36A	0.1343	0.8031	0.7798	0.055*	
H36B	0.0679	0.7177	0.7484	0.055*	
C37	−0.0454 (2)	0.76366 (17)	0.84885 (9)	0.0518 (4)	
H37A	0.0216	0.6980	0.8715	0.062*	
H37B	−0.0639	0.8282	0.8779	0.062*	
C38	−0.1988 (3)	0.74368 (17)	0.84541 (10)	0.0560 (5)	
H38A	−0.1793	0.6737	0.8217	0.067*	
H38B	−0.2535	0.7356	0.8936	0.067*	
C39	−0.3007 (2)	0.83800 (17)	0.80619 (9)	0.0476 (4)	
H39A	−0.3310	0.9061	0.8332	0.057*	
H39B	−0.3966	0.8199	0.8020	0.057*	
C40	−0.21765 (18)	0.85977 (15)	0.73291 (8)	0.0398 (4)	
H40A	−0.1981	0.7948	0.7041	0.048*	
H40B	−0.2853	0.9248	0.7100	0.048*	
C41	0.37181 (16)	0.85823 (12)	0.36844 (7)	0.0304 (3)	
C42	0.50058 (17)	0.89865 (13)	0.35564 (8)	0.0350 (3)	
H42	0.5251	0.9326	0.3920	0.042*	
C43	0.59281 (19)	0.88949 (13)	0.29019 (9)	0.0408 (4)	
H43	0.6808	0.9166	0.2812	0.049*	
C44	0.5541 (2)	0.84021 (13)	0.23855 (8)	0.0414 (4)	
C45	0.4275 (2)	0.80065 (14)	0.24890 (8)	0.0418 (4)	
H45	0.4034	0.7676	0.2120	0.050*	
C46	0.33557 (18)	0.81000 (13)	0.31451 (8)	0.0368 (3)	
H46	0.2472	0.7833	0.3227	0.044*	
C47	0.25088 (16)	0.58364 (11)	0.52907 (7)	0.0289 (3)	
C48	0.16850 (18)	0.50752 (13)	0.54914 (8)	0.0374 (3)	
H48	0.0833	0.5099	0.5268	0.045*	
C49	0.2099 (2)	0.42799 (15)	0.60171 (9)	0.0446 (4)	
H49	0.1529	0.3760	0.6156	0.054*	
C50	0.3341 (2)	0.42435 (13)	0.63383 (9)	0.0411 (4)	
H50	0.3629	0.3698	0.6699	0.049*	
C51	0.41641 (18)	0.50015 (13)	0.61351 (8)	0.0379 (3)	
H51	0.5017	0.4974	0.6360	0.046*	
C52	0.37666 (17)	0.58023 (12)	0.56091 (8)	0.0341 (3)	
H52	0.4343	0.6318	0.5469	0.041*	

### Atomic displacement parameters (Å^2^)

**Table:**

	*U*^11^	*U*^22^	*U*^33^	*U*^12^	*U*^13^	*U*^23^
S1	0.02799 (17)	0.02660 (17)	0.02791 (17)	−0.00381 (13)	−0.00096 (13)	−0.00506 (13)
F1	0.0534 (6)	0.0455 (5)	0.0388 (5)	−0.0197 (4)	−0.0164 (4)	0.0151 (4)
O1	0.0299 (5)	0.0274 (5)	0.0303 (5)	−0.0052 (4)	0.0018 (4)	−0.0032 (4)
O2	0.0408 (6)	0.0461 (6)	0.0321 (6)	−0.0108 (5)	−0.0057 (5)	−0.0099 (5)
C1	0.0247 (6)	0.0263 (6)	0.0279 (7)	−0.0058 (5)	−0.0032 (5)	−0.0034 (5)
C2	0.0239 (6)	0.0252 (6)	0.0313 (7)	−0.0060 (5)	−0.0039 (5)	−0.0054 (5)
C3	0.0253 (7)	0.0272 (6)	0.0305 (7)	−0.0054 (5)	−0.0030 (5)	−0.0040 (5)
C4	0.0268 (7)	0.0273 (7)	0.0337 (7)	−0.0069 (5)	−0.0061 (6)	−0.0044 (6)
C5	0.0281 (7)	0.0265 (7)	0.0415 (8)	−0.0026 (5)	−0.0016 (6)	−0.0063 (6)
C6	0.0283 (7)	0.0298 (7)	0.0379 (8)	−0.0050 (6)	0.0038 (6)	−0.0070 (6)
C7	0.0280 (7)	0.0276 (7)	0.0305 (7)	−0.0092 (5)	−0.0013 (6)	−0.0041 (5)
C8	0.0256 (6)	0.0275 (6)	0.0300 (7)	−0.0078 (5)	−0.0023 (5)	−0.0057 (5)
C9	0.0277 (7)	0.0257 (6)	0.0342 (7)	−0.0013 (5)	−0.0052 (6)	−0.0027 (6)
C10	0.0769 (13)	0.0317 (8)	0.0348 (9)	0.0002 (8)	−0.0135 (8)	−0.0024 (7)
C11	0.0766 (14)	0.0425 (10)	0.0307 (8)	0.0114 (9)	−0.0045 (9)	0.0003 (7)
C12	0.0541 (12)	0.0775 (15)	0.0591 (13)	−0.0066 (11)	0.0084 (10)	0.0296 (11)
C13	0.0655 (13)	0.0606 (12)	0.0668 (13)	−0.0370 (10)	−0.0172 (11)	0.0214 (10)
C14	0.0540 (10)	0.0463 (9)	0.0511 (10)	−0.0245 (8)	−0.0179 (8)	0.0088 (8)
C15	0.0307 (7)	0.0294 (7)	0.0271 (7)	−0.0121 (5)	−0.0047 (6)	−0.0021 (5)
C16	0.0290 (7)	0.0339 (7)	0.0310 (7)	−0.0107 (6)	−0.0031 (6)	−0.0004 (6)
C17	0.0317 (7)	0.0342 (7)	0.0366 (8)	−0.0101 (6)	−0.0097 (6)	0.0008 (6)
C18	0.0420 (8)	0.0339 (7)	0.0308 (7)	−0.0180 (6)	−0.0135 (6)	0.0049 (6)
C19	0.0412 (8)	0.0439 (8)	0.0290 (7)	−0.0176 (7)	−0.0008 (6)	0.0008 (6)
C20	0.0330 (8)	0.0356 (8)	0.0326 (8)	−0.0082 (6)	0.0002 (6)	−0.0029 (6)
C21	0.0252 (7)	0.0286 (7)	0.0314 (7)	−0.0030 (5)	−0.0011 (6)	−0.0032 (6)
C22	0.0381 (8)	0.0441 (9)	0.0333 (8)	−0.0102 (7)	−0.0009 (6)	0.0006 (7)
C23	0.0537 (11)	0.0563 (11)	0.0508 (11)	−0.0228 (9)	0.0069 (9)	0.0048 (9)
C24	0.0481 (10)	0.0618 (12)	0.0690 (13)	−0.0306 (9)	0.0061 (9)	−0.0120 (10)
C25	0.0454 (10)	0.0648 (12)	0.0546 (11)	−0.0243 (9)	−0.0049 (8)	−0.0156 (9)
C26	0.0376 (8)	0.0459 (9)	0.0339 (8)	−0.0125 (7)	−0.0034 (7)	−0.0053 (7)
S2	0.03003 (18)	0.03542 (19)	0.02744 (17)	−0.01091 (14)	−0.00264 (14)	−0.00723 (14)
F2	0.0748 (8)	0.0594 (7)	0.0381 (5)	−0.0195 (6)	0.0184 (5)	−0.0014 (5)
O3	0.0320 (5)	0.0322 (5)	0.0297 (5)	−0.0106 (4)	−0.0051 (4)	0.0005 (4)
O4	0.0364 (6)	0.0465 (6)	0.0418 (6)	−0.0160 (5)	−0.0130 (5)	−0.0062 (5)
C27	0.0259 (7)	0.0339 (7)	0.0276 (7)	−0.0093 (6)	−0.0040 (5)	−0.0036 (6)
C28	0.0242 (6)	0.0297 (7)	0.0295 (7)	−0.0066 (5)	−0.0072 (5)	−0.0039 (5)
C29	0.0264 (7)	0.0296 (7)	0.0314 (7)	−0.0092 (5)	−0.0031 (6)	−0.0040 (6)
C30	0.0243 (6)	0.0328 (7)	0.0313 (7)	−0.0067 (5)	−0.0039 (5)	−0.0052 (6)
C31	0.0308 (7)	0.0305 (7)	0.0352 (8)	−0.0059 (6)	−0.0046 (6)	−0.0080 (6)
C32	0.0339 (7)	0.0269 (7)	0.0397 (8)	−0.0091 (6)	−0.0071 (6)	−0.0022 (6)
C33	0.0252 (6)	0.0315 (7)	0.0290 (7)	−0.0075 (5)	−0.0060 (5)	0.0018 (5)
C34	0.0255 (7)	0.0342 (7)	0.0302 (7)	−0.0080 (6)	−0.0086 (6)	−0.0018 (6)
C35	0.0310 (7)	0.0343 (7)	0.0297 (7)	−0.0102 (6)	−0.0006 (6)	−0.0088 (6)
C36	0.0388 (9)	0.0591 (11)	0.0320 (8)	−0.0042 (8)	−0.0046 (7)	−0.0016 (7)
C37	0.0555 (11)	0.0612 (11)	0.0321 (8)	−0.0095 (9)	−0.0051 (8)	0.0021 (8)
C38	0.0765 (14)	0.0604 (11)	0.0371 (9)	−0.0361 (10)	0.0025 (9)	0.0002 (8)
C39	0.0418 (9)	0.0705 (12)	0.0381 (9)	−0.0308 (9)	0.0032 (7)	−0.0094 (8)
C40	0.0342 (8)	0.0537 (10)	0.0348 (8)	−0.0185 (7)	−0.0033 (6)	−0.0026 (7)
C41	0.0284 (7)	0.0340 (7)	0.0276 (7)	−0.0080 (6)	−0.0053 (6)	0.0028 (6)
C42	0.0327 (7)	0.0376 (8)	0.0346 (8)	−0.0109 (6)	−0.0047 (6)	0.0002 (6)
C43	0.0368 (8)	0.0404 (8)	0.0431 (9)	−0.0142 (7)	0.0018 (7)	0.0031 (7)
C44	0.0475 (9)	0.0381 (8)	0.0294 (8)	−0.0076 (7)	0.0057 (7)	0.0041 (6)
C45	0.0527 (10)	0.0447 (9)	0.0278 (7)	−0.0130 (7)	−0.0085 (7)	−0.0002 (7)
C46	0.0376 (8)	0.0451 (9)	0.0299 (7)	−0.0146 (7)	−0.0088 (6)	0.0026 (6)
C47	0.0267 (7)	0.0310 (7)	0.0288 (7)	−0.0080 (5)	−0.0006 (5)	−0.0089 (6)
C48	0.0342 (8)	0.0464 (9)	0.0379 (8)	−0.0196 (7)	−0.0065 (6)	−0.0050 (7)
C49	0.0510 (10)	0.0462 (9)	0.0440 (9)	−0.0268 (8)	−0.0065 (8)	0.0017 (7)
C50	0.0508 (10)	0.0372 (8)	0.0349 (8)	−0.0111 (7)	−0.0083 (7)	−0.0026 (7)
C51	0.0363 (8)	0.0395 (8)	0.0395 (8)	−0.0074 (6)	−0.0125 (7)	−0.0091 (7)
C52	0.0319 (7)	0.0346 (7)	0.0398 (8)	−0.0134 (6)	−0.0057 (6)	−0.0087 (6)

### Geometric parameters (Å, º)

**Table:**

S1—O2	1.4832 (11)	S2—O4	1.4955 (11)
S1—C1	1.7681 (13)	S2—C27	1.7589 (14)
S1—C21	1.8020 (15)	S2—C47	1.7948 (15)
F1—C18	1.3541 (16)	F2—C44	1.3546 (18)
O1—C7	1.3778 (17)	O3—C34	1.3687 (17)
O1—C8	1.3821 (16)	O3—C33	1.3801 (17)
C1—C8	1.3649 (19)	C27—C34	1.363 (2)
C1—C2	1.4457 (18)	C27—C28	1.4504 (19)
C2—C7	1.3917 (19)	C28—C33	1.3931 (19)
C2—C3	1.394 (2)	C28—C29	1.3959 (19)
C3—C4	1.3948 (19)	C29—C30	1.3960 (19)
C3—H3	0.9500	C29—H29	0.9500
C4—C5	1.409 (2)	C30—C31	1.401 (2)
C4—C9	1.510 (2)	C30—C35	1.515 (2)
C5—C6	1.384 (2)	C31—C32	1.388 (2)
C5—H5	0.9500	C31—H31	0.9500
C6—C7	1.380 (2)	C32—C33	1.378 (2)
C6—H6	0.9500	C32—H32	0.9500
C8—C15	1.4602 (19)	C34—C41	1.467 (2)
C9—C10	1.518 (2)	C35—C40	1.524 (2)
C9—C14	1.526 (2)	C35—C36	1.527 (2)
C9—H9	1.0000	C35—H35	1.0000
C10—C11	1.524 (2)	C36—C37	1.532 (2)
C10—H10A	0.9900	C36—H36A	0.9900
C10—H10B	0.9900	C36—H36B	0.9900
C11—C12	1.515 (3)	C37—C38	1.515 (3)
C11—H11A	0.9900	C37—H37A	0.9900
C11—H11B	0.9900	C37—H37B	0.9900
C12—C13	1.512 (3)	C38—C39	1.512 (3)
C12—H12A	0.9900	C38—H38A	0.9900
C12—H12B	0.9900	C38—H38B	0.9900
C13—C14	1.527 (2)	C39—C40	1.530 (2)
C13—H13A	0.9900	C39—H39A	0.9900
C13—H13B	0.9900	C39—H39B	0.9900
C14—H14A	0.9900	C40—H40A	0.9900
C14—H14B	0.9900	C40—H40B	0.9900
C15—C20	1.398 (2)	C41—C46	1.393 (2)
C15—C16	1.398 (2)	C41—C42	1.396 (2)
C16—C17	1.385 (2)	C42—C43	1.386 (2)
C16—H16	0.9500	C42—H42	0.9500
C17—C18	1.377 (2)	C43—C44	1.376 (2)
C17—H17	0.9500	C43—H43	0.9500
C18—C19	1.375 (2)	C44—C45	1.372 (2)
C19—C20	1.384 (2)	C45—C46	1.388 (2)
C19—H19	0.9500	C45—H45	0.9500
C20—H20	0.9500	C46—H46	0.9500
C21—C22	1.379 (2)	C47—C48	1.383 (2)
C21—C26	1.387 (2)	C47—C52	1.389 (2)
C22—C23	1.395 (3)	C48—C49	1.383 (2)
C22—H22	0.9500	C48—H48	0.9500
C23—C24	1.378 (3)	C49—C50	1.378 (2)
C23—H23	0.9500	C49—H49	0.9500
C24—C25	1.381 (3)	C50—C51	1.380 (2)
C24—H24	0.9500	C50—H50	0.9500
C25—C26	1.381 (2)	C51—C52	1.385 (2)
C25—H25	0.9500	C51—H51	0.9500
C26—H26	0.9500	C52—H52	0.9500
			
O2—S1—C1	106.57 (6)	O4—S2—C27	108.81 (6)
O2—S1—C21	107.16 (7)	O4—S2—C47	106.56 (6)
C1—S1—C21	97.93 (6)	C27—S2—C47	100.12 (6)
C7—O1—C8	106.71 (10)	C34—O3—C33	106.37 (11)
C8—C1—C2	107.55 (12)	C34—C27—C28	106.99 (12)
C8—C1—S1	128.04 (11)	C34—C27—S2	121.59 (11)
C2—C1—S1	123.92 (10)	C28—C27—S2	131.29 (11)
C7—C2—C3	119.91 (12)	C33—C28—C29	119.07 (13)
C7—C2—C1	104.85 (12)	C33—C28—C27	104.63 (12)
C3—C2—C1	135.24 (13)	C29—C28—C27	136.27 (13)
C2—C3—C4	118.67 (13)	C28—C29—C30	119.00 (13)
C2—C3—H3	120.7	C28—C29—H29	120.5
C4—C3—H3	120.7	C30—C29—H29	120.5
C3—C4—C5	119.18 (13)	C29—C30—C31	119.30 (13)
C3—C4—C9	120.78 (13)	C29—C30—C35	121.13 (13)
C5—C4—C9	119.98 (12)	C31—C30—C35	119.57 (12)
C6—C5—C4	123.00 (13)	C32—C31—C30	123.01 (13)
C6—C5—H5	118.5	C32—C31—H31	118.5
C4—C5—H5	118.5	C30—C31—H31	118.5
C7—C6—C5	116.03 (13)	C33—C32—C31	115.71 (13)
C7—C6—H6	122.0	C33—C32—H32	122.1
C5—C6—H6	122.0	C31—C32—H32	122.1
O1—C7—C6	126.08 (13)	C32—C33—O3	125.28 (13)
O1—C7—C2	110.72 (11)	C32—C33—C28	123.87 (13)
C6—C7—C2	123.19 (13)	O3—C33—C28	110.84 (12)
C1—C8—O1	110.16 (12)	C27—C34—O3	111.17 (12)
C1—C8—C15	135.74 (13)	C27—C34—C41	134.11 (13)
O1—C8—C15	114.09 (12)	O3—C34—C41	114.62 (12)
C4—C9—C10	113.60 (12)	C30—C35—C40	112.57 (12)
C4—C9—C14	110.00 (12)	C30—C35—C36	112.15 (12)
C10—C9—C14	111.43 (14)	C40—C35—C36	110.28 (13)
C4—C9—H9	107.2	C30—C35—H35	107.2
C10—C9—H9	107.2	C40—C35—H35	107.2
C14—C9—H9	107.2	C36—C35—H35	107.2
C9—C10—C11	111.83 (14)	C35—C36—C37	111.74 (14)
C9—C10—H10A	109.3	C35—C36—H36A	109.3
C11—C10—H10A	109.3	C37—C36—H36A	109.3
C9—C10—H10B	109.3	C35—C36—H36B	109.3
C11—C10—H10B	109.3	C37—C36—H36B	109.3
H10A—C10—H10B	107.9	H36A—C36—H36B	107.9
C12—C11—C10	111.41 (17)	C38—C37—C36	111.32 (15)
C12—C11—H11A	109.3	C38—C37—H37A	109.4
C10—C11—H11A	109.3	C36—C37—H37A	109.4
C12—C11—H11B	109.3	C38—C37—H37B	109.4
C10—C11—H11B	109.3	C36—C37—H37B	109.4
H11A—C11—H11B	108.0	H37A—C37—H37B	108.0
C13—C12—C11	111.13 (16)	C39—C38—C37	111.42 (15)
C13—C12—H12A	109.4	C39—C38—H38A	109.3
C11—C12—H12A	109.4	C37—C38—H38A	109.3
C13—C12—H12B	109.4	C39—C38—H38B	109.3
C11—C12—H12B	109.4	C37—C38—H38B	109.3
H12A—C12—H12B	108.0	H38A—C38—H38B	108.0
C12—C13—C14	110.90 (17)	C38—C39—C40	111.69 (15)
C12—C13—H13A	109.5	C38—C39—H39A	109.3
C14—C13—H13A	109.5	C40—C39—H39A	109.3
C12—C13—H13B	109.5	C38—C39—H39B	109.3
C14—C13—H13B	109.5	C40—C39—H39B	109.3
H13A—C13—H13B	108.0	H39A—C39—H39B	107.9
C9—C14—C13	111.28 (14)	C35—C40—C39	111.48 (13)
C9—C14—H14A	109.4	C35—C40—H40A	109.3
C13—C14—H14A	109.4	C39—C40—H40A	109.3
C9—C14—H14B	109.4	C35—C40—H40B	109.3
C13—C14—H14B	109.4	C39—C40—H40B	109.3
H14A—C14—H14B	108.0	H40A—C40—H40B	108.0
C20—C15—C16	118.54 (13)	C46—C41—C42	119.38 (14)
C20—C15—C8	119.35 (13)	C46—C41—C34	121.44 (13)
C16—C15—C8	122.09 (13)	C42—C41—C34	119.18 (13)
C17—C16—C15	120.69 (14)	C43—C42—C41	120.32 (15)
C17—C16—H16	119.7	C43—C42—H42	119.8
C15—C16—H16	119.7	C41—C42—H42	119.8
C18—C17—C16	118.55 (14)	C44—C43—C42	118.51 (15)
C18—C17—H17	120.7	C44—C43—H43	120.7
C16—C17—H17	120.7	C42—C43—H43	120.7
F1—C18—C19	118.68 (14)	F2—C44—C45	118.84 (16)
F1—C18—C17	118.49 (14)	F2—C44—C43	118.30 (15)
C19—C18—C17	122.83 (14)	C45—C44—C43	122.86 (15)
C18—C19—C20	118.03 (14)	C44—C45—C46	118.41 (15)
C18—C19—H19	121.0	C44—C45—H45	120.8
C20—C19—H19	121.0	C46—C45—H45	120.8
C19—C20—C15	121.35 (14)	C45—C46—C41	120.52 (15)
C19—C20—H20	119.3	C45—C46—H46	119.7
C15—C20—H20	119.3	C41—C46—H46	119.7
C22—C21—C26	121.79 (14)	C48—C47—C52	120.39 (14)
C22—C21—S1	118.64 (12)	C48—C47—S2	116.89 (11)
C26—C21—S1	119.36 (11)	C52—C47—S2	122.14 (11)
C21—C22—C23	118.21 (16)	C47—C48—C49	120.16 (14)
C21—C22—H22	120.9	C47—C48—H48	119.9
C23—C22—H22	120.9	C49—C48—H48	119.9
C24—C23—C22	120.53 (17)	C50—C49—C48	119.84 (15)
C24—C23—H23	119.7	C50—C49—H49	120.1
C22—C23—H23	119.7	C48—C49—H49	120.1
C23—C24—C25	120.37 (17)	C49—C50—C51	119.87 (15)
C23—C24—H24	119.8	C49—C50—H50	120.1
C25—C24—H24	119.8	C51—C50—H50	120.1
C24—C25—C26	120.06 (17)	C50—C51—C52	121.04 (14)
C24—C25—H25	120.0	C50—C51—H51	119.5
C26—C25—H25	120.0	C52—C51—H51	119.5
C25—C26—C21	119.04 (16)	C51—C52—C47	118.70 (14)
C25—C26—H26	120.5	C51—C52—H52	120.6
C21—C26—H26	120.5	C47—C52—H52	120.6
			
O2—S1—C1—C8	129.92 (13)	O4—S2—C27—C34	−110.88 (12)
C21—S1—C1—C8	−119.46 (13)	C47—S2—C27—C34	137.62 (12)
O2—S1—C1—C2	−41.04 (13)	O4—S2—C27—C28	64.38 (15)
C21—S1—C1—C2	69.59 (12)	C47—S2—C27—C28	−47.12 (14)
C8—C1—C2—C7	−0.03 (15)	C34—C27—C28—C33	0.14 (15)
S1—C1—C2—C7	172.51 (10)	S2—C27—C28—C33	−175.64 (11)
C8—C1—C2—C3	179.17 (15)	C34—C27—C28—C29	−177.66 (15)
S1—C1—C2—C3	−8.3 (2)	S2—C27—C28—C29	6.6 (3)
C7—C2—C3—C4	0.5 (2)	C33—C28—C29—C30	−0.6 (2)
C1—C2—C3—C4	−178.65 (14)	C27—C28—C29—C30	176.98 (15)
C2—C3—C4—C5	−1.3 (2)	C28—C29—C30—C31	1.4 (2)
C2—C3—C4—C9	175.88 (12)	C28—C29—C30—C35	−178.37 (12)
C3—C4—C5—C6	0.9 (2)	C29—C30—C31—C32	−0.7 (2)
C9—C4—C5—C6	−176.23 (14)	C35—C30—C31—C32	179.06 (13)
C4—C5—C6—C7	0.2 (2)	C30—C31—C32—C33	−0.8 (2)
C8—O1—C7—C6	179.79 (14)	C31—C32—C33—O3	−177.75 (13)
C8—O1—C7—C2	−0.35 (15)	C31—C32—C33—C28	1.6 (2)
C5—C6—C7—O1	178.77 (13)	C34—O3—C33—C32	178.94 (13)
C5—C6—C7—C2	−1.1 (2)	C34—O3—C33—C28	−0.50 (15)
C3—C2—C7—O1	−179.12 (12)	C29—C28—C33—C32	−1.0 (2)
C1—C2—C7—O1	0.24 (15)	C27—C28—C33—C32	−179.23 (13)
C3—C2—C7—C6	0.7 (2)	C29—C28—C33—O3	178.49 (11)
C1—C2—C7—C6	−179.89 (13)	C27—C28—C33—O3	0.22 (15)
C2—C1—C8—O1	−0.18 (15)	C28—C27—C34—O3	−0.47 (16)
S1—C1—C8—O1	−172.32 (10)	S2—C27—C34—O3	175.81 (9)
C2—C1—C8—C15	−178.81 (15)	C28—C27—C34—C41	175.71 (14)
S1—C1—C8—C15	9.0 (2)	S2—C27—C34—C41	−8.0 (2)
C7—O1—C8—C1	0.33 (15)	C33—O3—C34—C27	0.60 (15)
C7—O1—C8—C15	179.28 (11)	C33—O3—C34—C41	−176.38 (11)
C3—C4—C9—C10	40.31 (19)	C29—C30—C35—C40	−58.93 (18)
C5—C4—C9—C10	−142.58 (15)	C31—C30—C35—C40	121.35 (15)
C3—C4—C9—C14	−85.36 (17)	C29—C30—C35—C36	66.13 (18)
C5—C4—C9—C14	91.75 (16)	C31—C30—C35—C36	−113.59 (16)
C4—C9—C10—C11	−178.30 (15)	C30—C35—C36—C37	178.43 (14)
C14—C9—C10—C11	−53.4 (2)	C40—C35—C36—C37	−55.25 (19)
C9—C10—C11—C12	54.3 (2)	C35—C36—C37—C38	55.3 (2)
C10—C11—C12—C13	−56.0 (2)	C36—C37—C38—C39	−54.7 (2)
C11—C12—C13—C14	56.8 (2)	C37—C38—C39—C40	55.0 (2)
C4—C9—C14—C13	−178.92 (15)	C30—C35—C40—C39	−178.71 (14)
C10—C9—C14—C13	54.2 (2)	C36—C35—C40—C39	55.21 (18)
C12—C13—C14—C9	−55.9 (2)	C38—C39—C40—C35	−55.58 (19)
C1—C8—C15—C20	−169.42 (16)	C27—C34—C41—C46	42.2 (2)
O1—C8—C15—C20	11.98 (18)	O3—C34—C41—C46	−141.70 (14)
C1—C8—C15—C16	12.2 (2)	C27—C34—C41—C42	−137.88 (17)
O1—C8—C15—C16	−166.43 (12)	O3—C34—C41—C42	38.20 (18)
C20—C15—C16—C17	0.4 (2)	C46—C41—C42—C43	−0.9 (2)
C8—C15—C16—C17	178.83 (13)	C34—C41—C42—C43	179.18 (14)
C15—C16—C17—C18	−0.4 (2)	C41—C42—C43—C44	0.2 (2)
C16—C17—C18—F1	−178.38 (12)	C42—C43—C44—F2	−179.64 (14)
C16—C17—C18—C19	0.6 (2)	C42—C43—C44—C45	0.6 (3)
F1—C18—C19—C20	178.28 (13)	F2—C44—C45—C46	179.63 (14)
C17—C18—C19—C20	−0.7 (2)	C43—C44—C45—C46	−0.6 (3)
C18—C19—C20—C15	0.7 (2)	C44—C45—C46—C41	−0.2 (2)
C16—C15—C20—C19	−0.5 (2)	C42—C41—C46—C45	0.9 (2)
C8—C15—C20—C19	−178.99 (14)	C34—C41—C46—C45	−179.20 (14)
O2—S1—C21—C22	−10.24 (14)	O4—S2—C47—C48	25.64 (13)
C1—S1—C21—C22	−120.38 (12)	C27—S2—C47—C48	138.89 (12)
O2—S1—C21—C26	174.99 (11)	O4—S2—C47—C52	−163.09 (12)
C1—S1—C21—C26	64.85 (13)	C27—S2—C47—C52	−49.83 (13)
C26—C21—C22—C23	−0.6 (2)	C52—C47—C48—C49	0.7 (2)
S1—C21—C22—C23	−175.20 (13)	S2—C47—C48—C49	172.11 (13)
C21—C22—C23—C24	0.9 (3)	C47—C48—C49—C50	−0.4 (3)
C22—C23—C24—C25	−0.9 (3)	C48—C49—C50—C51	0.1 (3)
C23—C24—C25—C26	0.6 (3)	C49—C50—C51—C52	−0.2 (2)
C24—C25—C26—C21	−0.2 (3)	C50—C51—C52—C47	0.5 (2)
C22—C21—C26—C25	0.2 (2)	C48—C47—C52—C51	−0.7 (2)
S1—C21—C26—C25	174.83 (13)	S2—C47—C52—C51	−171.72 (11)

### Hydrogen-bond geometry (Å, º)

Cg1 and Cg2 are the centroids of the C41–C46 4-fluorophenyl ring and the C2–C7 benzene ring, respectively.

**Table:**

*D*—H···*A*	*D*—H	H···*A*	*D*···*A*	*D*—H···*A*
C10—H10*B*···O2^i^	0.99	2.47	3.384 (2)	153
C22—H22···O2^i^	0.95	2.42	3.2365 (19)	144
C19—H19···O4^ii^	0.95	2.44	3.3442 (19)	159
C40—H40*B*···*Cg*1^iii^	0.99	2.84	3.763 (2)	155
C45—H45···*Cg*2	0.95	2.82	3.602 (2)	140
C50—H50···*Cg*1^iv^	0.95	2.80	3.619 (2)	144

Symmetry codes: (i) −*x*+1, −*y*+1, −*z*; (ii) −*x*, −*y*+1, −*z*+1; (iii) −*x*, −*y*+2, −*z*+1; (iv) −*x*+1, −*y*+1, −*z*+1.
